# Neurologic Injury and Brain Growth in the Setting of Long-Gap Esophageal Atresia Perioperative Critical Care: A Pilot Study

**DOI:** 10.3390/brainsci9120383

**Published:** 2019-12-17

**Authors:** Samuel S. Rudisill, Jue T. Wang, Camilo Jaimes, Chandler R. L. Mongerson, Anne R. Hansen, Russell W. Jennings, Dusica Bajic

**Affiliations:** 1 Department of Anesthesiology, Critical Care and Pain Medicine, Boston Children’s Hospital, Boston, MA 02115, USA; srudisil@alumni.nd.edu (S.S.R.); jue.wang@childrens.harvard.edu (J.T.W.); chandler.mongerson@childrens.harvard.edu (C.R.L.M.); 2 Department of Anaesthesia, Harvard Medical School, Boston, MA 02115, USA; 3 Department of Radiology, Division of Neuroradiology, Boston Children’s Hospital, and Department of Radiology, Harvard Medical School, Boston, MA 02115, USA; Camilo.JaimesCobos@childrens.harvard.edu; 4 Department of Pediatrics, Division of Neonatal Medicine, Boston Children’s Hospital, and Department of Pediatrics, Harvard Medical School, Boston, MA 02115, USA; anne.hansen@childrens.harvard.edu; 5 Department of Surgery, Boston Children’s Hospital, and Department of Surgery, Harvard Medical School, Boston, MA 02115, USA; russell.jennings@childrens.harvard.edu; 6 Esophageal and Airway Treatment Center, Boston Children’s Hospital, Boston, MA 02115, USA

**Keywords:** infant, LGEA, midazolam, morphine, MRI, neuroimaging, opioids, term, tolerance, weaning

## Abstract

We previously showed that infants born with long-gap esophageal atresia (LGEA) demonstrate clinically significant brain MRI findings following repair with the Foker process. The current pilot study sought to identify any pre-existing (PRE-Foker process) signs of brain injury and to characterize brain and corpus callosum (CC) growth. Preterm and full-term infants (*n* = 3/group) underwent non-sedated brain MRI twice: *before* (PRE-Foker scan) and *after* (POST-Foker scan) completion of perioperative care. A neuroradiologist reported on qualitative brain findings. The research team quantified intracranial space, brain, cerebrospinal fluid (CSF), and CC volumes. We report novel qualitative brain findings in preterm and full-term infants born with LGEA *before* undergoing Foker process. Patients had a unique hospital course, as assessed by secondary clinical end-point measures. Despite increased total body weight and absolute intracranial and brain volumes (cm^3^) between scans, normalized brain volume was decreased in 5/6 patients, implying delayed brain growth. This was accompanied by both an absolute and relative CSF volume increase. In addition to qualitative findings of CC abnormalities in 3/6 infants, normative CC size (% brain volume) was consistently smaller in all infants, suggesting delayed or abnormal CC maturation. A future larger study group is warranted to determine the impact on the neurodevelopmental outcomes of infants born with LGEA.

## 1. Introduction

In humans, the period between the third trimester of gestation and the second year of life represents a critical phase of neurodevelopment with long-term implications for cognitive and behavioral function [1,2,3]. The importance of this phase is emphasized by dramatic brain growth, synaptogenesis, and myelination, with cognitive and motor development following accordingly. This critical period represents a window of vulnerability during which exposure to environmental insult can alter the developmental trajectory, resulting in potential consequences ranging from delayed acquisition of function to severe neurological disorder [4,5].

Children born with congenital anomalies/critical illness find themselves at an early disadvantage. Coexisting congenital abnormalities and greater severity of illness are associated with worse neurological outcomes [6,7,8]. Several studies have identified various aspects of intensive care to have potential neurodevelopmental effects of their own. The need to undergo major surgical procedures presents a source of significant pain in addition to metabolic, hemodynamic, and respiratory stress [9]. Previous reports revealed abnormalities in pain processing, neurodevelopment, and behavior following repeated invasive procedures [10]. While administration of analgesics may reduce the stress associated with pain and discomfort, prolonged sedation leads to the development of tolerance and dependence if administered for ≥5 days, requiring subsequent weaning and a prolonged hospital stay [11,12]. 

Additionally, infections affecting infants while in the neonatal intensive care unit have been linked to a range of abnormalities in brain development and increased risk of long-term neurological sequelae [13]. Risk of infection can be decreased by breastfeeding [14], but critically ill infants may require parenteral nutrition that does not confer the same developmental advantages as breastmilk [15]. Although each of these individual stressors and risk factors have been studied in animals and/or humans, their cumulative/additive impact on neurodevelopment remains to be determined. 

We previously showed clinically significant MRI findings [16,17], increased cerebrospinal fluid (CSF) volume [16] and globally decreased brain size [18] in critically ill premature and full-term infants *after* long-gap esophageal atresia (LGEA) repair using the Foker process [19,20,21]. Interestingly, none of the patients studied had any previously known brain findings (e.g., presence of cranial ultrasound findings) or neurological symptoms that would implicate neurological disease (e.g., seizures). LGEA is a rare birth defect characterized by a gap in the esophagus that is too large to be repaired by direct anastomosis. Without repair, it is impossible for infants to feed by mouth or swallow secretions, making them respectively prone to inadequate growth and infections such as pneumonia. LGEA represents a unique type of esophageal atresia [22]. Compared to short-gap esophageal atresia, LGEA is more likely to be an isolated defect and associated with trisomy 21. Further, LGEA is less commonly found with VACTERL anomalies (vertebral, anorectal, cardiac, tracheo-esophageal fistula and/or esophageal atresia, renal, and limb defects/malformations) relative to short-gap esophageal atresia. However, the revolutionary Foker process encourages the natural growth and lengthening of infant’s existing esophagus pouches, with the end result being an intact esophagus, but requires two separate thoracotomies with a subsequent prolonged postoperative intubation [16,17]. Thus, the objective of this pilot study was to compare matched MRIs from patients undergoing the Foker process, with the first scan obtained, “PRE-Foker,” to reflect pre-existing signs of brain injury, and the second scan obtained “POST-Foker,” after a period of perioperative critical care, to determine the effects of the surgical and medical care in the intervening period. Similarly to our previous reports [16,17,18], none of the patients studied in this pilot had any previously known neurological findings. In addition to qualitative MRI findings, we report on quantitative measurements of brain and corpus callosum (CC) growth, and CSF compartment status. Our secondary clinical end-point measures were body weight (kg) at PRE- and POST-Foker process brain MRI scans, the number of anesthesia events, cumulative exposure to minimum alveolar concentration (MAC) equivalent hours of anesthetic agents, length of intubation (as a proxy of postoperative sedation with opioids and benzodiazepines), weaning of sedation, as well as length of muscle relaxant, antibiotic, steroid and total parenteral nutrition (TPN) administration (days).

## 2. Materials and Methods

### 2.1. Study Design and Subjects

As an extension of our previous work [16,17,18], this pilot study was approved by the Boston Children’s Hospital Review Board as a ‘no more than minimal risk’ study. Informed written consent was obtained from parents before participation in accordance with the Declaration of Helsinki and Good Clinical Practice. Patients were eligible to participate if they were less than 12 months old and were selected to undergo the Foker process [19,20,21] for treatment of LGEA. Exclusion criteria included: (1) any known neurological disease (e.g., seizures); (2) cranial ultrasound findings (e.g., ventricular enlargement, hemorrhage); (3) chromosomal abnormalities (e.g., Down’s Syndrome); (4) prenatal drug exposure; (5) extreme prematurity (<28 weeks GA); and/or (6) MRI incompatible implants. Subjects were categorized into moderate-to-late preterm [born 28- to 36 weeks gestational age (GA)] and full-term (born 37- to 42 weeks GA) infants. Complex perioperative care included several stages [23]: (1) Foker I thoracotomy to place traction sutures onto blind esophageal ends; (2) Esophageal lengthening, which uses continuous traction on the esophagus to induce esophageal growth; (3) Foker II thoracotomy to approximate esophageal ends and perform primary esophageal anastomosis; (4) Post-Foker healing of the anastomosis. To facilitate postoperative mechanical ventilation, infants underwent prolonged sedation (≥5 days), that led to pharmacological dependence to sedative agents [12,24,25] requiring subsequent weaning from sedation medications (viz. opioids and benzodiazepines). Although we did not analyze potential symptoms of withdrawal or withdrawal prevention management (for Review on weaning treatment, see [26]), we confirmed that extended administration of opioids and benzodiazepines was indicated specifically for weaning management as per primary team and/or pain service consult notes. In this pilot case report, subjects were categorized and named as Preterm 1, 2, 3 and Term 1, 2, 3 in order of increasing corrected age at time of post-Foker MRI scan. Baseline demographics and clinical information for each patient are displayed in Table 1 and Table 2.

### 2.2. MRI Acquisition

All infants underwent two non-sedated research brain MRIs in a 3T TrioTim MRI system equipped with a 32 channel, receive-only head coil and body-transmission (Siemens Healthcare Inc., Malvern, PA, USA): (1) just prior to undergoing surgery for LGEA repair (PRE-Foker scan) and (2) after completion of postoperative sedation and weaning treatment (POST-Foker scan).

#### 2.2.1. Preparation for Non-Sedated MRI

To improve infants’ compliance during neuroimaging sessions in the research setting, we used the ‘feed and wrap’ approach [27,28,29,30] to support natural sleep, as previously reported by our group [16]. Briefly, imaging was done during infants’ sleeping time (evenings and nights), and parents were encouraged to perform sleep routines (e.g., nursing, rocking, singing, etc.). Both foam earplugs (Newmatic Medical, Birmingham, AL, USA) and earmuffs (MRI-Safe Neonatal Noise Guards, Universal Medical, Norwood, MA, USA) were placed for noise protection, while the sides of the head were padded with sponges and soft sheets. All infants were continuously monitored for heart rate and oxygen saturation. One of the parents/guardians was allowed to stay with the infant in the imaging suite throughout MRI acquisition. 

#### 2.2.2. MRI Sequences

As previously reported [16,18], structural T2-weighted axial images were acquired using FSE sequence [TR/TE = 12624/110 ms; FA = 120°; FOV = 180 × 180 mm^2^; voxels = 0.35 × 0.35 mm^2^; 63 slices, 2 mm thickness] and were used to obtain intracranial, brain and CSF volumes. T1-weighted images were acquired using MPRAGE sequence [TR/TE = 2520/1.74 ms; FA = 7°; FOV = 192 × 192 mm^2^; voxels = 1 × 1 × 1 mm^3^; 144 sagittal slices] and were used to obtain brain and CC volumes [31,32].

### 2.3. Qualitative MRI Analysis

A pediatric neuroradiologist on call reviewed MRI scans for any findings of clinical significance, which were subsequently also checked by Dr. C. Jaimes. Comparisons were made between PRE- and POST-Foker MRI scans for each patient to assess qualitative changes in brain morphology over the treatment course. All clinically relevant findings, as well as patient age (months) and weight at scan (kg), are summarized in Table 3.

### 2.4. Quantitative MRI Analysis

#### 2.4.1. T2-Weighted Brain MRI Tissue Segmentation

Structural segmentation of T2-weighted images using Morphologically Adaptive Neonatal Tissue Segmentation (MANTiS) toolbox [33] was adapted as previously described by our group [16,18] to allow for intracranial, brain and CSF volume analysis in older infants. FMRIB Software Library (FSL; v.5.0) tools were also used for preprocessing and post-segmentation editing of older infants’ scans. In summary, analysis involved the following steps:

- **Preprocessing:** (i) Intracranial space segmentation: T2 images were skull-stripped using the unvalidated ‘Simple Watershed Scalping’ module in the MANTiS toolbox, followed by manual editing in FSLview; (ii) Bias field correction was performed using FMRIB’s Automated Segmentation Tool (FAST) [34]; (iii) Setting the image origin employed the ‘Origin to the Center of Mass’ module in the MANTiS toolbox;
- **MANTiS Segmentation:** Preprocessed images underwent MANTiS segmentation pipeline [33]. Automated CSF segmentations were visually inspected and subsequently edited to correct for any tissue misclassifications as described below;
- **Post-Segmentation Editing:** Automated CSF segmentations were (i) masked to zero voxels outside of intracranial space, (ii) thresholded at 40% to eliminate voxels with <40% probability of representing CSF, and (iii) converted to binary masks. Additional complex editing was undertaken due to the frequent exclusion of CSF spaces and inclusion of brain tissue. Specifically, the partial volume estimate map of CSF generated by FAST [34] was (i) thresholded at 50% to eliminate voxels with <50% of their volume comprising CSF, (ii) converted to a binary mask, and (iii) combined with subject’s thresholded/binarized MANTiS CSF mask for each case. Consequently, FAST’s CSF map effectively filled in spaces missing in MANTiS’ CSF segmentation (e.g., cisterns, 4th ventricle, and sulcal spaces), resulting in a ‘comprehensive’ CSF image. Additional minor manual editing was performed to erase misclassified brain tissue. A single individual performed all manual editing to ensure consistent tissue delineation. This final total CSF segmentation was further divided into extra-axial and ventricular spaces by manually erasing ventricular CSF from the total CSF segmentation to produce extra-axial space segmentation.

#### 2.4.2. T1-Weighted Brain MRI Tissue Segmentation

Evaluation of corpus callosum (CC) volumes from T1-weighted images required preprocessing that included alignment along the anterior commissure–posterior commissure line using Freeview (v.2.0) to correct for any head tilt during MRI acquisition, as illustrated in our previous reports (Figure 2 in [16]). Subsequently, images were manually segmented to delineate 3-D CC and to facilitate whole brain extraction using ITK-SNAP software [35] (v.3.6.0; www.itksnap.org) as previously reported [32] and summarized below:

- **Whole Brain Segmentation.** Semi-automated brain tissue segmentation was achieved as follows: (i) Skull-stripping of T1 images by manually tracing the brain outline (includes ventricles); and (ii) Partial volume segmentation of CSF using FAST [34]. Using tools in FSL, CSF partial volume estimate maps were (a) thresholded at 99% to eliminate voxels with <99% of their volume comprising CSF, (b) converted to binary masks, and (c) subtracted from the masks of brain outline (includes ventricles) in order to generate masks of brain tissue that exclude the ventricles. The latter brain-only masks subsequently underwent additional (d) minor manual editing to draw in any missing brain tissue.
- **Manual CC Segmentation.** We extended available protocol for CC segmentation by Yu et al. [36], as previously described by our group [32]. Effectively, CC was traced as an arching line from the tips of the lateral ventricles to the in-folding of the cingulate gyrus. We used both a neuroanatomical atlas [37] and a fiber tract-based atlas of human white matter [38] for anatomical referencing to guide total CC segmentation.

#### 2.4.3. MRI Data Volumetry 

ITK-SNAP [35] volume estimation tool was used to obtain volumes, which were reported as absolute (cm^3^) and normalized (% of intracranial volume (%ICV) or % of total CSF (%CSF)) values to correct for inter-individual variation [39]. The difference between the T2-weighted intracranial and total CSF volumes represented brain volume. Similarly, the difference between total CSF and extra-axial space volumes represented ventricular space volume. Normalized values for T1-weighted CC volume were calculated as % of brain volume, since total brain tissue is appropriate for understanding how CC size changes with respect to the brain as a whole [40]. 

### 2.5. Quantification of Pharmacological Treatment

We have previously illustrated the representative perioperative time- and event-course for the repair of LGEA requiring the Foker process [16,17]. In this pilot study, we quantified several clinical end-point measures. Both the number of anesthetic events and cumulative exposure to minimum alveolar concentration (MAC) equivalent hours of anesthetic agents were obtained from digital anesthesia records (AIMS Charts, 2019 Citrix Receiver Application, v. 19.3.0.21). The duration of anesthesia events that occurred at outside hospitals *prior* to the Foker process were estimated (i.e., endotracheal intubation = 0.25 h; placement of central access line = 0.5 h; laparoscopic gastrostomy placement = 1 h), likely resulting in the underestimation of total anesthesia exposure. Additional clinical data of interest were obtained from the electronic medical record, Powerchart^R^ (Cerner, London, UK) and included length (in days) of (1) intubation as a proxy of postoperative sedation with opioids and benzodiazepines, and (2) weaning of sedation, as well as days of administration of (3) muscle relaxants, (4) antibiotics, (5) steroids, and (6) TPN with fat emulsion throughout the course of treatment. We also listed the type of drug administered for muscle relaxants, sedation, antibiotics, and steroids. Regarding sedation medication administration, we distinguished between (i) length of intubation (as a proxy of postoperative sedation with opioids and benzodiazepines), (ii) weaning of sedation following extubation that included opioids and benzodiazepines, and (iii) weaning of sedation treatment, limited to the use of alpha-2 agonists (clonidine and/or dexmedetomidine).

## 3. Results

We present three preterm and three full-term infants treated with the Foker process requiring complex perioperative critical care. Baseline demographics and clinical information that includes anesthetic and surgical information PRE- and POST-Foker scans are summarized in Table 1. All patients were admitted to Boston Children’s Hospital with a primary diagnosis of LGEA. Preterm 2, Term 2, and Term 3 patient also had a tracheoesophageal fistula (TEF). Preterm 3 presented with pulmonary hypertension (pHTN), intrauterine growth restriction (IUGR), and failure to thrive (FTT). Term 3 was also diagnosed with tetralogy of Fallot (TOF). None of the patients had VACTERL association (vertebral, anorectal, cardiac, TEF and/or EA, renal, and limb defects/malformations).

### 3.1. Perioperative Critical Care for LGEA

#### 3.1.1. Pre-Foker Management

Prior to the PRE-Foker process MRI scan, each patient had previously undergone at least one intervention requiring anesthesia administration (Table 1). A summary of procedural events prior to PRE-Foker process brain MRI scan is shown in Table 2. The majority were diagnostic (viz. computer tomography (CT) scan; esophago-duodenoscopy), simple procedures (viz. endotracheal intubation, peripherally inserted central catheter (PICC) placement), and/or minor surgeries (e.g., gastrostomy placement). Only Term 1 underwent single simple anesthesia exposure for CT scan. Preterm 2 and Term 2 underwent previous thoracotomies for unsuccessful LGEA repair, while Term 2 and 3 had undergone major cardiac surgeries—patch repair of heart valves and TOF repair, respectively. Of note, Term 3 had a history of cardiac arrest requiring resuscitation. 

#### 3.1.2. Anesthesia Management for the Foker Process.

Shortly after hospital admission, each patient underwent the staged repair of LGEA with Foker process that allowed for native esophageal tissue growth. Anesthesia management was provided for Foker I (placement of traction sutures) and Foker II thoracotomies (esophageal end-to-end anastomosis). The anesthetic management for each thoracotomy was comprised of general anesthesia utilizing both inhalational and intravenous agents. Maintenance of anesthesia was achieved with infusions of dexmedetomidine, fentanyl, and vecuronium, as well as one half MAC inhaled sevoflurane. During surgical dissection around airway structures, the anesthesia team performed continuous bronchoscopy in order to guide surgical repair by helping surgeons visualize the anatomy. The fluid management goals were to keep the patients euvolemic with judicious use of crystalloids in order to maintain a mean arterial pressure between 50–85 mmHg. Postoperatively, patients were taken, intubated, sedated, and paralyzed to the intensive care unit in order to allow for the growth and healing of the esophagus with traction sutures (Foker I) or after end-to-end anastomosis (Foker II).

In addition to the Foker process, some of the patients required additional anesthesia administration, mostly for esophago-duodenoscopy, to evaluate the healing of the esophageal anastomosis, and esophageal dilations as needed. A summary of cumulative exposure to MAC equivalent anesthesia *between* PRE- and POST-Foker process MRI scans is reported for each patient in Table 1.

#### 3.1.3. Complex Critical Care Management

**Figure 1:**
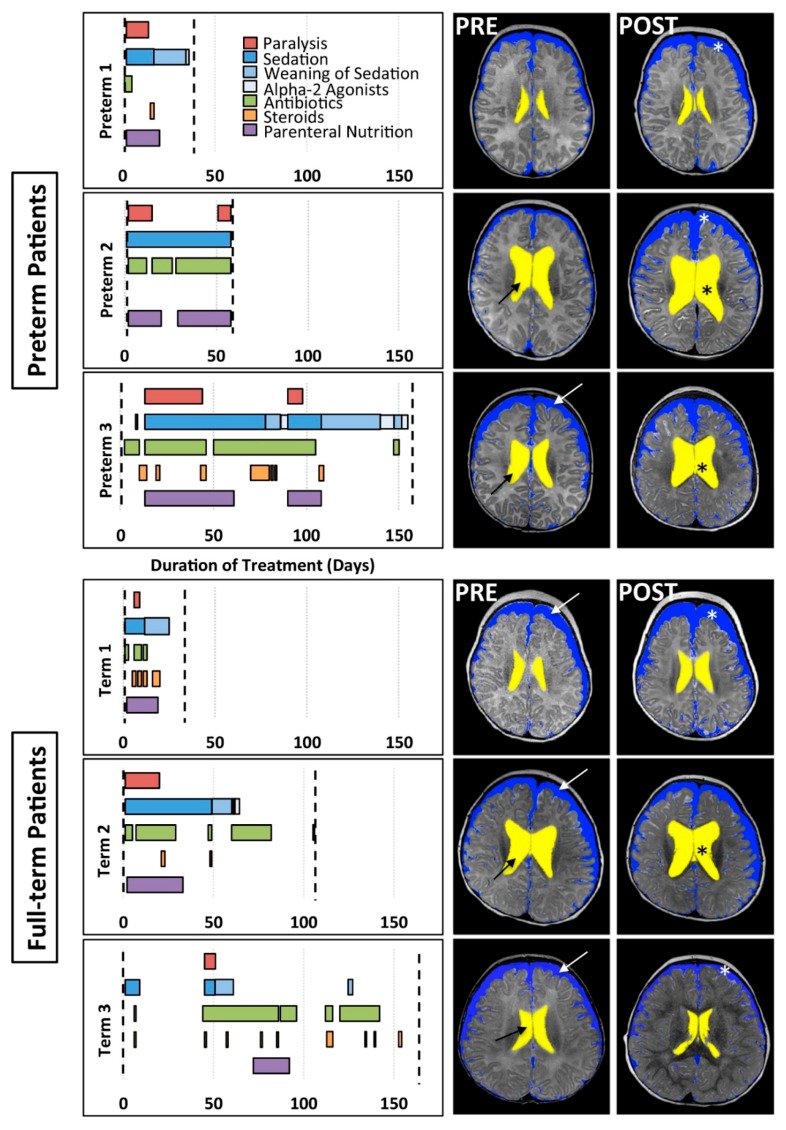
Pharmacological course (graphs) and corresponding representative brain MRI cross-sections (right panels). Graphs illustrate the timing and duration of various pharmacological treatments for preterm (*n* = 3) and full-term (*n* = 3) patients between PRE- and POST-Foker brain MRI scans (vertical dashed lines). The POST-Foker process brain MRI scan for Preterm 2 was obtained before completion of sedation weaning due to transfer to another hospital. Representative T2-weighted images in axial view (at the level of the body of the lateral ventricles) illustrate brain parenchyma and segmentation masks for divisions of CSF: extra-axial space (blue) and ventricles (yellow). Qualitative evaluation of the PRE-Foker process brain MRI scans showed increased CSF volumes in extra-axial space (white arrows) and/or ventricles (black arrows) for all except Preterm 1. Furthermore, POST-Foker process scans showed a mild increase in CSF in either or both CSF compartments (*) for all subjects, including a case of novel subdural hematoma (Preterm 1, POST-Foker scan; obscured by the blue mask; see also Figure 2 in [17]).

As evidenced from Figure 1 graphs, each individual patient had a unique clinical course.

- **Postoperative Intubation.** Postoperative sedation in combination with immobilization served to preserve the temporary placement of surgical devices, facilitate proper tissue expansion, and allow for safe healing following both Foker I and Foker II thoracotomies. Length of intubation served as a proxy of length of sedation. Combinations of opioids (viz. morphine) and benzodiazepines (viz. midazolam) were used for primary sedation management and were administered as both infusions and intermittent boluses per institutional guidelines [12]. Preterms 1, 2, 3, and Term 1 additionally received fentanyl boluses, and lorazepam was administered to Preterm 2 and Term 1 during this sedation period.
- **Weaning from Opioid and Benzodiazepine Sedation.** Because each infant received prolonged sedation (≥5 days), which is associated with the development of physical dependence [12,24,25], weaning of sedation was required following extubation. Morphine, midazolam, and lorazepam were used to facilitate this process, though Term 1 did not receive lorazepam and Term 2 did not receive midazolam. Preterm 3 also received oxycodone during weaning. The administration of α-2 adrenergic agonists (clonidine and/or dexmedetomidine), initiated during primary sedation, was continued into the weaning period in all patients except Term 1 and Term 3. Only Preterm 2 did not have complete drug evaluation for sedation weaning, due to transfer to another hospital before completion of treatment.
- **Other.** As graphically illustrated in Figure 1, in addition to muscle relaxants (red bars), sedative medications for intubation (dark blue bars), and weaning of sedation medications (lighter blue bars), a variety of antibiotics were prescribed to each patient to prevent/treat infection throughout the clinical course (green bars). All patients except Preterm 2 also periodically received steroids (orange bars). Due to the nature of the illness, nutritional resources were supplied by parenteral route (purple bars).

### 3.2. Qualitative Clinical Brain MRI Reports

#### 3.2.1. PRE-Foker Process Brain MRI 

Although no patient had any known neurological concerns prior to treatment of the LGEA, PRE-Foker treatment brain MRI revealed abnormalities in both preterm and full-term patients (Table 3). Specifically, extra-axial space was enlarged in Preterm 1 and Preterm 3, while Preterm 2, Preterm 3, Term 1, and Term 2 displayed enlarged lateral ventricles (Figure 1). As outlined in Table 3, CC abnormalities were reported for Preterm 3, Term 1, and Term 2. Additional findings reported included widened Sylvian fissures (Preterm 1), intraventricular hemorrhage (Preterm 2), vascular anomaly (Preterm 3), and chronic subdural collection (Term 1). Despite the most challenging clinical course (including cardiac arrests and cardiac surgery for TOF), Term 3 only exhibited prominent subarachnoid spaces on the PRE-Foker treatment scan.

#### 3.2.2. POST-Foker Process Brain MRI 

In all cases, pre-existing (viz. PRE-Foker process) brain MRI findings were exacerbated and/or novel findings were reported following the perioperative course, as indicated by the POST-Foker process brain MRI reports (Figure 1 and Table 3). Specifically, Preterm 1 developed subdural hematoma (see also Figure 2 in [17]), Preterm 2 experienced an arterial ischemic/hemorrhagic stroke, and Term 1 showed the progressive enlargement of CSF spaces (for latter, see also Figure 5 in [16]). Both Term 2 and Term 3 exhibited increased extra-axial space following treatment; Term 3 further demonstrated brachycephaly and under-operculation of the Sylvian fissures.

### 3.3. Quantitative Brain MRI Analysis

#### 3.3.1. Brain and Total CSF Volume Change 

**Figure 2:**
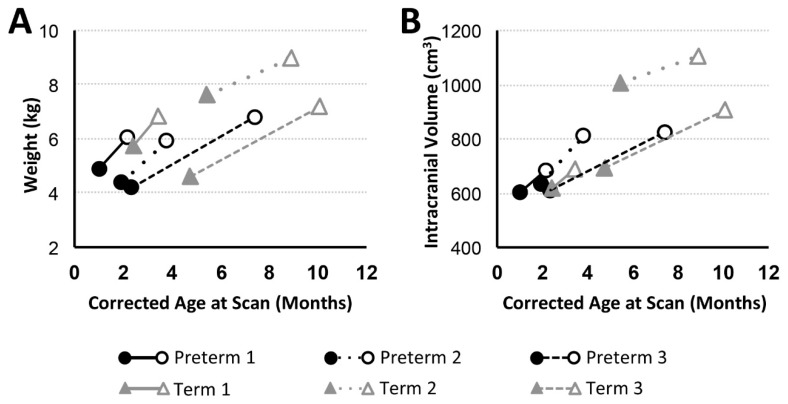
Body Weight and Intracranial Volume with Advancing Age. Graphs show body weight (kg; (**A**)) and intracranial volume (cm^3^; (**B**)) trajectories for preterm (*n* = 3; black circles) and full-term (*n* = 3; gray triangles) patients between PRE- (filled marker) and POST- (open marker) Foker process brain MRI scans. Both preterm and full-term infants show an increase in weight and intracranial volume (an indirect marker of head circumference) between the two MRI scans.

**Figure 3:**
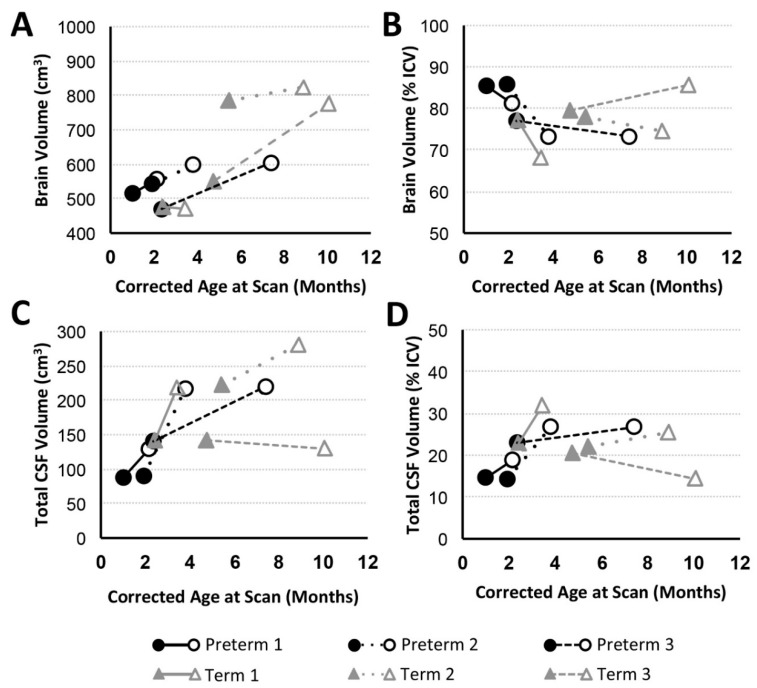
Brain and Total cerebrospinal fluid (CSF) Volume with Advancing Age. Graphs display absolute and normalized brain (**A**,**B**) and total CSF (**C**,**D**) volume trajectories for preterm (*n* = 3; black circles) and full-term (*n* = 3; gray triangles) patients between PRE- (filled marker) and POST- (open marker) Foker process brain MRI scans. Despite brain growth in 5/6 infants (**A**; similar results found for T1-weighted analysis), this growth was not proportional to intracranial volume (**ICV**; Figure 2B), resulting in decreased normalized brain volumes (**B**). Reciprocal changes are reported for total absolute (**C**) and normalized CSF (**D**) volumes.

In addition to general weight gain (Figure 2A), growth was indicated by patterns of increasing intracranial volume (a proxy for head circumference; Figure 2B) in both preterm and full-term infants between MRI scans. Similarly, all patients except Term 1 also experienced brain growth during the time period between the two scans (see absolute brain volumes in Figure 3A for T2-weighted and the last figure for T1-weighted analyses). Quantitative analysis of CSF confirmed increased absolute volumes of total CSF for all patients except Term 3 (Figure 3C). In contrast, normalized values (%intracranial volume) of total brain volume decreased and those of CSF increased (except Term 3), implicating delayed or attenuated brain growth and increased CSF volume during treatment (Figure 3). The lack of increased CSF for Term 3 accounts for more normative % values despite the most complicated clinical course, and novel incidental findings on POST-Foker process MRI scan (Table 2 and Table 3).

#### 3.3.2. CSF Compartments Volume Change

**Figure 4:**
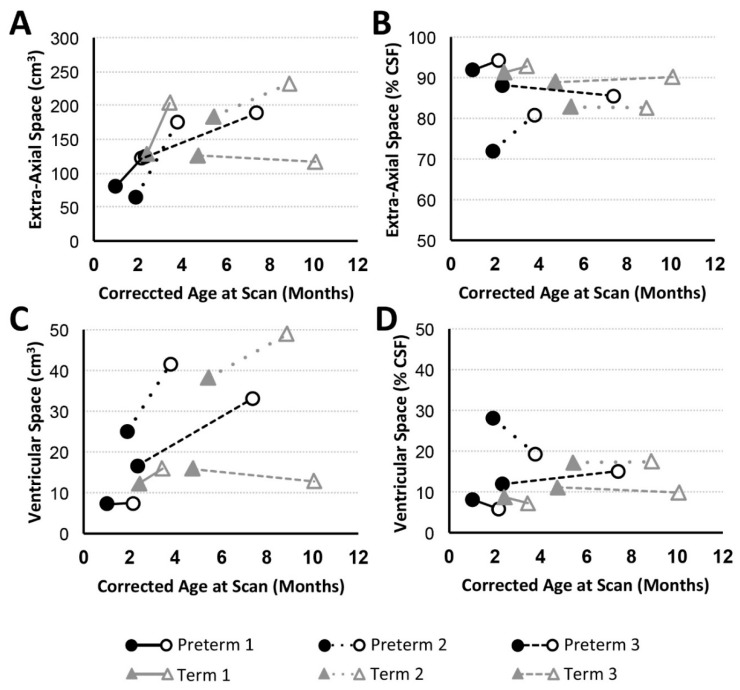
Volumes of CSF Compartments with Advancing Age. Graphs display absolute and normalized extra-axial space (**A**,**B**) and ventricular (**C**,**D**) volume trajectories for preterm (*n* = 3; black circles) and full-term (*n* = 3; gray triangles) patients between PRE- (filled marker) and POST- (open marker) Foker process brain MRI scans. Based on T2-weighted analysis, all patients (except Term 3) showed increase in absolute extra-axial space (**A**) and ventricular (**C**) volumes, similar to the pattern observed for absolute total CSF (Figure 3C). Normalized volumes as % total CSF volume) are shown in Panels (**B**,**D**).

Volumetric analysis of CSF divisions revealed an absolute increase in both extra-axial space and ventricular volumes for all patients except Term 3, which showed a subtle decrease (Figure 4A,C). Variation in normalized values (as a % total CSF; Figure 4B,D) reflects a more subtle expansion of either extra-axial space (Preterm 1, Preterm 2, Term 1), ventricles (Preterm 3), or both compartments (Term 2).

#### 3.3.3. Corpus Callosum Volume Change

**Figure 5:**
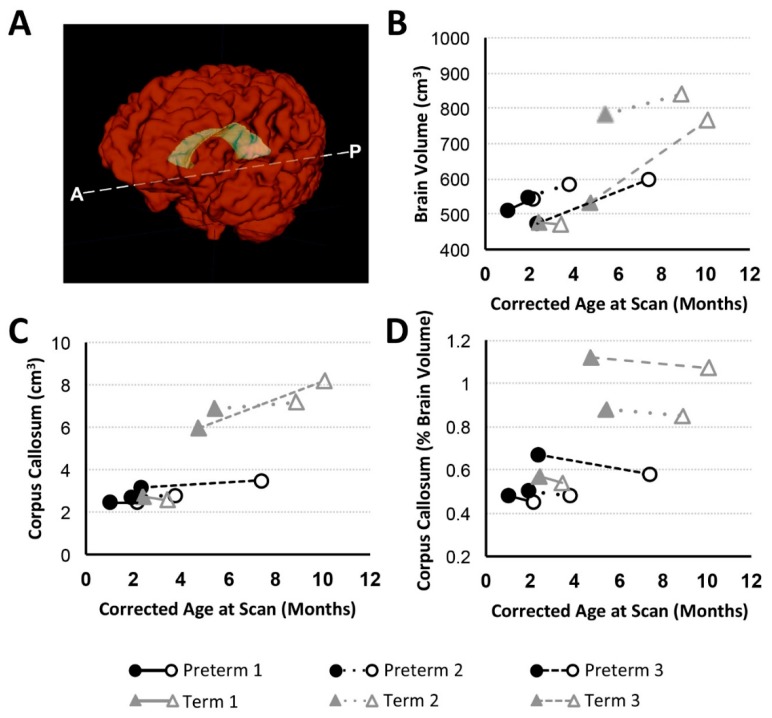
Total Brain Volume and Corpus Callosum with Age. Panel A shows 3-D renderings of total brain (red) and corpus callosum (CC; yellow) structural masks based on T1-weighted brain MRI segmentation. Graphs illustrate volumetric data for preterm (*n* = 3; black circles) and full-term (*n* = 3; gray triangles) patients at PRE- (filled marker) and POST- (open marker) Foker brain MRI. Panels (**B**) and (**C**) show absolute brain and CC volume (cm^3^), respectively. Normalized volume of CC (as %brain volume) is shown in Panel (**D**).

We report CC growth (Figure 5C) during a time period between PRE- and POST-Foker MRI scans in all patients except Preterm 1 (no change) and Term 1 (slight decrease). However, normalized CC values (as % brain volume) uniformly decreased between MRI scans in all subjects (Figure 5D), highlighting a reduced rate of corpus callosum growth relative to the whole brain during complex postoperative clinical care for LGEA with the Foker process.

## 4. Discussion

In this pilot study, we report qualitative and quantitative brain findings in preterm (*n* = 3) and full-term infants (*n* = 3) born with LGEA *prior* to undergoing the Foker process with complex perioperative critical care. None of the patients involved in this pilot study had any previously known neurological findings. Due to unique individual clinical presentations and variable previous histories, our findings raise previously unrecognized concerns regarding existing brain abnormalities prior to the Foker process. Despite increasing total body weight and intracranial volume (a proxy of head circumference) over the course of LGEA repair process, normalized total brain volume decreased in 5/6 patients, implying delayed brain growth during the perioperative critical care period for LGEA repair. Such a decrease in brain growth was accompanied by a relative and absolute increase in CSF volume. In addition to qualitative findings of CC abnormalities in 3/6 infants, normative CC size (% of brain volume) was reduced for all infants over time, implicating abnormal or delayed CC maturation.

### 4.1. PRE-Foker Process Brain MRI Findings in Infants Born with LGEA

This pilot study extends our previous reports [16,17] by providing additional evidence for the existence of *qualitative* brain abnormalities *prior* to Foker process repair in infants born with LGEA without any previously known neurological concerns. It is well known that up to half of all newborns with esophageal atresia have one or more other birth defects [41], but it still remains unknown if these involve intrinsic brain abnormalities. The brain abnormalities seen may be explained by the fact that the infants presented in this report received at least one previous intervention requiring anesthesia administration prior to the PRE-Foker MRI. Specifically, *qualitative* MRI findings in preterm infants (*n* = 3; Table 3) are noted following anesthesia exposure for diagnostic and simple surgical interventions (Table 2). Such findings are consistent with the body of knowledge related to the prevalence of brain abnormalities in premature infants [42,43,44,45]. Similarly, full-term infants presented in the current report show clinically significant PRE-Foker *qualitative* brain MRI findings (*n* = 3; Table 3) following simple diagnostic procedures (Term 1), thoracic non-cardiac surgery (Term 2) and cardiac repair with history of cardiac arrests (Term 3; see also Table 2). It is known that brain injury and lesions are reported in infants born with critical congenital heart defects [46,47,48]. However, a recent meta-analysis of functional MRI demonstrated that neurodevelopmental delay in infants with critical congenital heart disease is mainly from *prenatal* injury and not infant cardiac surgery with a cardiopulmonary bypass (see Review [49]). Therefore, our results call for future studies that would determine the intrinsic brain status of full-term infants born with LGEA in the absence of prior anesthesia exposure. Further research is warranted to better understand the potential mechanisms of brain injury and its time of onset. 

### 4.2. Impact of Complex Perioperative Critical Care for the Foker Process in Infants with LGEA

#### 4.2.1. Qualitative Brain MRI Findings

We also show that pre-existing *qualitative* brain abnormalities were exacerbated over the course of complex critical care (Figure 1; Table 3), similar to the previously reported literature evaluating stressors in the neonatal intensive care unit [50]. A recent study [7] reported that infants undergoing neonatal surgery for noncardiac congenital anomalies are at risk of postoperative brain injury (e.g., nonparenchymal abnormalities, including intraventricular and subdural hemorrhage), potentially accounting for the neurodevelopmental delay frequently observed in this population [8]. 

#### 4.2.2. Brain Growth in Infancy

Since brain growth is not proportional to intracranial volume change (Figure 2 and Figure 3), head circumference may not be a reliable index of brain growth in this patient cohort of infants with LGEA. We previously reported *quantitatively* smaller global brain volume [16,18] following completion of perioperative critical care (POST-Foker scan) in both premature and full-term infants with LGEA in comparison to otherwise healthy controls. In this pilot study, we find that brain growth (as % of ICV) decreased in 5/6 patients studied, suggesting delayed brain growth during perioperative critical care for LGEA repair (Figure 3). There is growing evidence in the literature to support the notion that infants with congenital gastrointestinal anomalies experience multiple stressors while hospitalized early in life [51] that may lead to altered brain growth patterns [52,53] and neurodevelopmental delay [8,54,55]. The predictive value of volumetric MRI analysis of early brain size for neurodevelopmental outcome has been shown [56,57,58]. Furthermore, extensive imaging studies in infants with congenital heart disease also reported perioperative brain injury [47,48] and delayed brain maturation [46,59], with the latter being predictive of poorer neurodevelopmental outcomes [6]. While several studies investigated the long-term neurological effects of major surgery, anesthesia exposure, and prolonged sedation on the developing brain [3,8,60,61,62,63,64,65,66], the cumulative immediate impact on brain morphology and growth in infants born with LGEA remains to be elucidated. Additionally, long-term neurodevelopmental follow-up is needed in this vulnerable population undergoing Foker process repair of LGEA.

#### 4.2.3. Increased CSF Volume in Infancy

As shown in Figure 4, variation in normalized CSF volume reflected the more subtle expansion of the extra-axial (Preterm 1, Preterm 2, Term 1), ventricular (Preterm 3) CSF, or both compartments (Term 1). Similar findings of increased extra-axial space (viz. idiopathic external hydrocephalus without evidence of ventricular enlargement or hydrocephalus) and ventricular enlargement were previously reported in infants with LGEA [16,17,67]. Such findings have also been described as part of a benign condition in the absence of surgery [68,69,70,71] and as part of external hydrocephalus not associated with cerebral atrophy [72]. Although no infants had VACTERL association (Table 1), hydrocephalus is a potential component of VACTERL (viz. VACTERL-H), which is now considered a distinct genetic entity with autosomal recessive or X-linked recessive inheritance [73,74,75,76]. Presence of enlarged CSF spaces on MRI was associated with poor short-term developmental outcomes in neonates treated with cardiopulmonary bypass [77]. Whether resulting from prematurity, reduction in adjacent brain tissue volume, or an increase in CSF itself [69,71,78], an increased volume of CSF spaces has been linked to moderate-to-severe disability [79,80] and long-term neurodevelopmental impairment [56,81]. The impact of increased CSF in infants with LGEA warrants future longitudinal neurobehavioral follow up.

#### 4.2.4. Corpus Callosum Maturation in Infancy

As the largest white matter tract in the brain, the CC demonstrates a particularly high incidence of anomaly and altered growth in the premature infant [82,83,84]. In this study, we report *qualitative* CC abnormalities in 1 preterm infant POST-Foker process repair (Preterm 3) and 2 full-term infants (Term 1 and Term 2) prior to LGEA repair (Table 2). White matter injury is considered the most common type of injury both before and after surgery and is associated with a less mature brain [85]. A similar report [7] describes parenchymal lesions in both full-term and preterm infants following surgery for gastrointestinal anomalies, many of which consisted of punctate white matter lesions. Although in this report we show that absolute CC volumes increased (except in Term 1, which showed a slight volume decrease over the course of treatment; Figure 5C), normalized CC values (% of brain volume) decreased in all cases (Figure 5D), indicating a disproportionately reduced rate of CC maturation relative to the whole brain. The short-term and long-term neurobehavioral impacts of these findings are not known. While one study of very preterm infants found no association between CC size and neurodevelopmental function at two year follow-up [84], others reported decreased CC growth predicted reduced motor performance at two years of age [83] as well as in later childhood [86]. Future studies in this vulnerable population of full-term and premature infants with LGEA should also include diffusion tensor imaging and tractography to shed light on the possible mechanisms underlying implied decreased white matter maturation and global brain growth.

### 4.3. Study Limitations

The findings presented in this study must be interpreted in the context of several limitations. (1) Pre-Existing Findings: Although infants with known brain abnormalities or neurologic disease were excluded from the study, no prenatal or postnatal MRI scans were available to rule out inherently altered brain structure in analyzed subjects. (2) Study Size: In the context of high variability of concomitant malformations, co-morbidities, and incidental brain MRI findings, further studies of larger cohorts are needed to support the presented findings in infants with LGEA and their relevance to surgery and critical care. (3) Sex Differences: Sex may be an important determinant of brain development that should be considered in future prospective large cohort studies. (4) Timing of the Brain MRI: A wide range of corrected age at the time of brain scans introduces a potential bias in this longitudinal pilot data collection. Any final interpretation insight with respect to brain and CC growth should await future prospective studies that include a uniform and narrower age range for PRE- and POST-Foker brain MRI scans.

## 5. Conclusions

Because there is evidence that brain injury can occur both in the pre- and postoperative periods, improved strategies to diagnose and prevent injury in these arenas are much needed and should be applied to infants with LGEA. Further investigation is warranted to identify neuroprotective management strategies for the operating room and intensive care unit settings to preserve neurologic function and optimize long-term neurodevelopmental outcomes in infants undergoing the complex Foker process for LGEA repair. 

## Figures and Tables

**Table 1:** Baseline Demographics and Clinical Information.

Baseline Demographics	Preterm 1	Preterm 2	Preterm 3	Term 1	Term 2	Term 3
Sex	F	M	F	F	M	M
Race	White	White	AA	AA	Hispanic	Other
Gestational age (weeks)	33	34	28	37	39	37
Birth weight (kg)	2.64	1.87	0.55	2.8	3.03	Unknown
Birth by cesarean section	No	No	Yes	Yes	Yes	Unknown
**Clinical Information**						
Initial Admission Service	NICU	NICU	MSICU	NICU	MSICU	MSICU
Main Diagnosis	LGEA	LGEA/TEF	LGEA	LGEA	LGEA/TEF	LGEA/TEF
Secondary Diagnoses	-	-	pHTN, IUGR, FTT	-	-	TOF
**Prior to PRE- Foker MRI Scan**						
Total number of Surgical Events	4	2	4	1	4	5
MAC Equivalent Anesthesia (h)	2.25	8.00	2.50	0.62	14.12	19.18
**Between PRE- and POST-Foker Scans**						
Total number of Surgical Events	3	8	13	3	8	17
MAC Equivalent Anesthesia (h)	15.90	34.30	56.38	17.53	30.55	27.80
Length of Muscle Relaxation (days)	12	20	39	3	19	6
Length of Intubation/Sedation (days)	15	57	83	11	48	14
Length of Weaning of Sedation (days)	17	*	44	13	16	12
Length of Antibiotic Treatment (days)	4	51	99	8	51	78
Length of Steroid Administration (days)	2	0	23	10	3	11
Length of TPN (days)	18	47	66	17	31	20

Table 1 summarizes baseline demographic and clinical data for preterm (*n* = 3) and full-term (*n* = 3) patients. Listed patient information is arranged according to increasing corrected age at POST-Foker process brain MRI. Durations of surgeries prior to PRE-Foker process brain MRI scan were estimated when performed at outside institution, as described in *Methods*. Clinical course for LGEA repair involved complex post-operative pharmacological treatment including prolonged sedation to facilitate postoperative mechanical ventilation. *Abbreviations*: AA, African American; F, female; FTT, failure to thrive; IUGR, intrauterine growth restriction; M, male; MAC, minimal-alveolar concentration; MRI, magnetic resonance imaging; MSICU, medical-surgical intensive care unit; NICU, neonatal intensive care unit; pHTN, pulmonary hypertension; TEF, tracheo-esophageal fistula; TOF, tetralogy of Fallot; TPN, total parenteral nutrition. (*) Could not be calculated due to patient transfer to the outside hospital before the end of weaning period.

**Table 2:** Clinical Events Prior to PRE-Foker Process Brain MRI Scan.

Patient	Event	Age at Event (months)	Anesthesia Exposure (h)	Notable Clinical Events/Procedures Performed
Preterm 1	1	0	0.25	Endotracheal Intubation
	2	0	0.50	PICC Insertion
	3	0.03	1.00	Laparoscopic Assisted G-tube Placement
	4	2.57	0.50	PICC Insertion
Preterm 2	1	0.07	4.00	Bronchoscopy, Thoracotomy, TEF Repair, Esophageal Immobilization
	2	2.43	4.00	Inguinal Hernia Repair with Diagnostic Laparoscopy, Circumcision, Tongue Tie Release, EGD
Preterm 3	1	1.93	1.00	Open Gastrostomy with Contrast Study via G-tube
	2	1.97	0.25	Endotracheal Intubation
	3	2.20	0.50	PICC Insertion
	4	3.50	0.75	Gastrostomy Revision with Fluoroscopy and G-tube Exchange
Term 1	1	3.07	0.62	Airway CT Scan
Term 2	1	0.03	6.00	Thoracotomy, TEF Repair, Chest Tube and G-tube Placement
	2	0.90	1.00	PICC Insertion
	3	3.07	6.00	Patch Repair of VSD, Suture Closure of ASD, PDA Ligation
	4	5.57	1.12	CT Scan
Term 3	1	0	1.00	Emergent Tracheostomy due to Cardiac Arrest following birth
	2	0.10	6.00	Thoracotomy for EA/TEF Repair, Tracheostomy Replacement, Gastrostomy
	3	5.10	7.70	TOF Repair, EGD, TEE, Direct Laryngoscopy and Bronchoscopy
	4	5.30	0	Cardiac Arrest due to clogged tracheostomy; Post-arrest Cooling
	5	5.47	1.70	Direct Laryngoscopy and Bronchoscopy
	6	5.50	2.78	Airway MRI

Table 2 summarizes notable procedural events requiring anesthesia administration prior to PRE-Foker brain MRI scan for preterm (n=3) and full-term (n=3) patients. Durations of anesthesia exposure were estimated for procedures performed outside of Boston Children’s Hospital as described in *Methods*. *Abbreviations*: ASD, atrial septal defect; CT, computerized tomography; EA, esophageal atresia; EGD, esophagoduodenoscopy; G-tube, gastrostomy tube; MRI, magnetic resonance imaging; PDA, patent ductus arteriosus; PICC, peripherally inserted central catheter; TEE, transesophageal echocardiography; TEF, tracheoesophageal fistula; TOF, tetralogy of Fallot; VSD, ventricular septal defect.

**Table 3:** Clinical Brain MRI Reports.

**PREMATURE PATIENTS**	**Preterm 1**		**Preterm 2**		**Preterm 3**	
MRI Scan	Pre-Rx	Post-Rx	Pre-Rx	Post-Rx	Pre-Rx	Post-Rx
Corrected Age at Scan (months)	1.02	2.16	1.93	3.80	2.36	7.41
Weight at Scan (kg)	4.910	6.040	4.380	5.920	4.200	6.810
***MRI Findings***	1. Increased Extra-Axial Space 2. Widened Sylvian Fissures	1. Increased Extra-Axial Space 2. Widened Sylvian Fissures 3. SDH	1. Enlarged Ventricles 2. IVH	1. Enlarged Ventricles 2. IVH 3. Increased Extra-Axial Space 4. Arterial Ischemic/Hemorrhagic Stroke	1. Increased Extra-Axial Space 2. Abnormality of CC 3. Enlarged Ventricles 4. Vascular Anomaly	1. Increased Extra-Axial Space 2. Abnormality of CC 3. Enlarged Ventricles 4. Vascular Anomaly
**FULL-TERM PATIENTS**	**Term 1**		**Term 2**		**Term 3**	
MRI Scan	Pre-Rx	Post-Rx	Pre-Rx	Post-Rx	Pre-Rx	Post-Rx
Corrected Age at Scan (months)	2.43	3.44	5.44	8.89	4.75	10.07
Weight at Scan (kg)	5.750	6.845	7.600	8.964	4.600	7.175
***MRI Findings***	1. Enlarged Ventricles 2. CC Thinning 3. Chronic Subdural Collection	1. Progressive Enlargement of CSF Spaces	1. Enlarged Ventricles 2. CC Thinning	1. Enlarged Ventricles 2. CC Thinning 3. Increased Extra-Axial Space	1. Prominent Extra-Axial Space	1. Increased Extra-Axial Space 2. Brachycephaly 3. Under Operculation of Sylvian Fissures

Table 3 summarizes patient information and clinically significant findings at PRE- (PRE-Rx) and POST-Foker process (POST-Rx) brain MRI scans. Patients are grouped according to preterm and full-term status (n=3/group) and organized by increasing corrected gestational age at the time of POST-Rx MRI scan. Text in blue denotes novel findings on POST-Rx brain scan. Abbreviations: CC, corpus callosum; IVH, intraventricular hemorrhage; SDH, subdural hematoma.

## Abbreviations

ASD	atrial septal defect
CC	corpus callosum
CSF	cerebrospinal fluid
CT	computerized tomography
EA	esophageal atresia
ECMO	extracorporeal membrane oxygenation
EGD	esophagoduodenoscopy
F	female
FAST	FMRIB’s Automated Segmentation Tool
FSL	FMRIB Software Library
FTT	failure to thrive
G-tube	gastrostomy tube
GA	gestational age
ICV	intracranial volume
IUGR	intrauterine growth restriction
LGEA	long-gap esophageal atresia
M	male
MAC	minimum alveolar concentration
MANTiS	morphologically adaptive neonatal tissue segmentation
MRI	magnetic resonance imaging
MSICU	medical-surgical intensive care unit
NICU	neonatal intensive care unit
PDA	patent ductus arteriosus
pHTN	pulmonary hypertension
PICC	peripherally inserted central catheter
Rx	treatment/therapy
SDH	subdural hematoma
TEE	transesophageal echocardiography
TEF	tracheoesophageal fistula
TOF	tetralogy of Fallot
TPN	total parenteral nutrition
VACTERL	Referring to vertebral, anorectal, cardiac, TEF and/or EA, renal, and limb malformations
VSD	ventricular septal defect
